# Limited transgenerational effects of environmental temperatures on thermal performance of a cold-adapted salmonid

**DOI:** 10.1093/conphys/coab021

**Published:** 2021-04-24

**Authors:** Chantelle M Penney, Gary Burness, Joshua K R Tabh, Chris C Wilson

**Affiliations:** 1 Environmental and Life Sciences Graduate Program, Trent University, Peterborough, Ontario K9J 7B8, Canada; 2 Department of Biology, Trent University, Peterborough, Ontario K9L 0G2, Canada; 3 Ontario Ministry of Natural Resources and Forestry, Trent University, Peterborough, Ontario K9L 0G2, Canada

**Keywords:** Climate change, critical thermal maximum, transgenerational plasticity, lake trout, thermal tolerance, epigenetic

## Abstract

The capacity of ectotherms to cope with rising temperatures associated with climate change is a significant conservation concern as the rate of warming is likely too rapid to allow for adaptative responses in many populations. Transgenerational plasticity (TGP), if present, could potentially buffer some of the negative impacts of warming on future generations. We examined TGP in lake trout to assess their inter-generational potential to cope with anticipated warming. We acclimated adult lake trout to cold (10°C) or warm (17°C) temperatures for several months, then bred them to produce offspring from parents within a temperature treatment (cold-acclimated and warm-acclimated parents) and between temperature treatments (i.e. reciprocal crosses). At the fry stage, offspring were also acclimated to cold (11°C) or warm (15°C) temperatures. Thermal performance was assessed by measuring their critical thermal maximum (CTM) and the change in metabolic rate during an acute temperature challenge. From this dataset, we also determined their resting and peak (highest achieved, thermally induced) metabolic rates. There was little variation in offspring CTM or peak metabolic rate, although cold-acclimated offspring from warm-acclimated parents exhibited elevated resting metabolic rates without a corresponding increase in mass or condition factor, suggesting that transgenerational effects can be detrimental when parent and offspring environments mismatch. These results suggest that the limited TGP in thermal performance of lake trout is unlikely to significantly influence population responses to projected increases in environmental temperatures.

## Teaser

Adult lake trout held at two temperatures were interbred to study the influence of parental thermal environments on the next generation’s thermal physiology. Offspring performance reflected both their own rearing environment and parental influences, although parental effects on offspring physiology were limited and not always beneficial.

## Introduction

Populations are being forced to respond to climate change as environmental temperatures continue to increase towards their viable limits (Hazen *et al*., 2013; Galbraith *et al*., 2014; Luo *et al*., 2015). Many species are resorting to migration and range shifts where movement to more suitable habitats is possible (e.g. freshwater fish, Chu *et al*., 2005; birds, VanDerWal *et al*., 2013; and mussels, Inoue & Berg, 2017), but those that are unable to relocate will need to acclimatize or adapt to warmer conditions if they are to persist. Organisms with little phenotypic plasticity may not be able to acclimatize to projected climatic conditions (Somero, 2010; Kelly *et al*., 2014), and the potential for rapid adaptive responses will be limited by the available standing genetic variation for traits under selection (Stockwell *et al*., 2003) and seems likely to be outpaced by rapidly changing environmental temperatures (Comte & Olden, 2017). This may be particularly challenging for core metabolic process such as thermal physiology, as rapid adaptation would require existing variation at the many genes underlying these pathways (Willi *et al*., 2006). In particular, populations that are small or isolated or have adapted to thermally stable habitats may be particularly vulnerable, as they are expected to have reduced standing genetic variation and less evolutionary potential (Willi *et al*., 2006; Meier *et al*., 2014). For species with long generation times, the rate of environmental change may also outpace the fixation of beneficial alleles (O’Grady *et al*., 2008; Visser, 2008; Munday *et al*., 2013). Within-generation and transgenerational plasticity (TGP) can potentially influence adaptation of populations to climate change by mitigating impacts of climate change-related stressors, providing more time for adaptation to occur (Bernatchez, 2016; Smith *et al*., 2016).

Populations may be able to compensate for long-term changes in temperature by preconditioning their offspring for harsher environments (Yin *et al*., 2019), which may, over time, influence adaptation (Bonduriansky *et al*., 2012). This preconditioning can involve maternal and paternal (non-genetic) effects including nutrient provisioning of the eggs, transfer of hormones and other cytoplasmic components and inheritance of epigenetic factors, which can change the way genes are expressed (Deans & Maggert, 2015; Charlesworth *et al*., 2017). This non-genetic inheritance can be observed through studies of TGP, which is a plastic response that occurs when the effects of the parent’s environment appear in the offspring’s phenotype (Bell & Hellmann, 2019). Though the occurrence and impact of transgenerational/anticipatory effects are still under debate (Uller *et al*., 2013; Yin *et al*., 2019; Sánchez-Tójar *et al*., 2020), TGP has been shown to benefit some fish species when faced with environmental warming, including three-spined stickleback (Shama *et al*., 2014), sheepshead minnow (Salinas & Munch, 2012) and tropical damselfish (Donelson *et al*., 2012; Munday *et al*., 2017). These fish are warm-adapted or eurythermal species, and it has not yet been confirmed whether TGP can benefit cold-adapted, stenothermal ectotherms. It is also unclear whether TGP is contingent on existing genetic variation, which is relevant to populations that have adapted to cold, stable environments since they are likely to have experienced reduced genetic variation over time (Willi *et al*. 2006; Wilson, 2017).

The lake trout (*Salvelinus namaycush*) is a cold-adapted, stenothermal salmonid (Martin & Olver, 1980; Casselman, 2008) under significant threat from climate change (Evans, 2007; Guzzo & Blanchfield, 2017). Populations of lake trout are restricted to northern oligotrophic lakes in North America, preferring temperatures between 10 and 12°C (Edsall & Cleland, 2000; Martinez *et al*., 2009). Lake trout habitat is transforming due to climate change: lake surface temperatures are increasing, the length of time that lakes are covered by ice is shortening and the extent of cool, highly oxygenated refuges are becoming limited during the summer (Reist *et al*., 2016; Guzzo & Blanchfield, 2017). These environmental changes already have an observable negative impact on lake trout as warmer temperatures at spawning reduces the survival of the fry at hatch (Casselman, 2002). Furthermore, evidence suggests that standing genetic variation is low for some populations of lake trout (Perrier *et al*., 2017), and there is little variation in the capacity for within-generation temperature acclimation within and among allopatric populations (McDermid *et al*., 2013; Kelly *et al*., 2014). The lake trout is an ideal model species to study whether TGP occurs in cold-adapted, stenothermal organisms, as their limited within-generation plasticity provides an opportunity to understand how TGP fits within the scope of possible thermal responses of organisms that are forced to cope with climate change.

We hypothesized that TGP occurs in lake trout, potentially enabling them to cope with warmer environments. Conversely, TGP may be limited or non-existent in lake trout, based on the species’ narrow temperature preference and thermal habitat stability (Martin & Olver, 1980; Evans, 2007; Wilson & Mandrak, 2004). To test these hypotheses, we acclimated hatchery raised, adult lake trout to cool (optimal, 10°C) and warm (17°C) temperatures, then used a full factorial mating design to cross fish within a temperature treatment (cold-acclimated pairs and warm-acclimated pairs) and between temperature treatments (i.e. reciprocal crosses). Their offspring were also acclimated to cool (11°C) and warm (15°C) temperatures so that offspring environments matched or mismatched that of their mother and/or father. This allowed us to observe transgenerational effects when offspring and parental environments matched and compare them with effects when temperature conditions differed between generations. Because the mothers and fathers were from matched or mismatched environments, this provided us with an opportunity to assess the relative parental contribution of the parents to offspring thermal performance. Given the evidence supporting anticipatory effects from both mothers and fathers (Marshall, 2015; Shama *et al*., 2016), we hypothesized that parents would additively contribute to TGP if it occurred.

**Figure 1:**
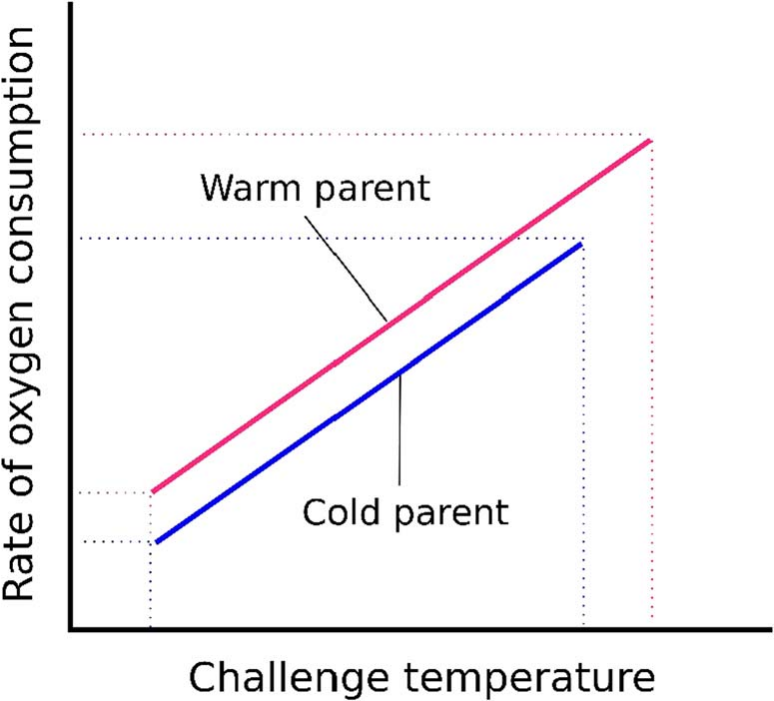
The predicted transgenerational effect of parental acclimation temperature on the rate of oxygen consumption of their offspring. The effect could be driven by either maternal or paternal acclimation temperatures or both. Interactions would be observed as a crossing of the lines. The resting and peak (highest achieved, thermally induced) rates of oxygen consumption are represented as the lowermost and uppermost ends of the lines.

We looked for evidence of TGP in the offspring’s upper thermal tolerance (measured as critical thermal maximum, CTM) and metabolic response (measured as the rate of O_2_ consumption, MO_2_) to an acute temperature increase. Our predictions were based on the evidence that standard metabolic rate in warm-acclimated adult lake trout would be elevated due to temperature acclimation (Kelly *et al*., 2014) and that this phenotype would be passed on to their offspring, predisposing them for a higher resting MO_2_. We also predicted that peak (highest achieved, thermally induced) MO_2_ would increase in offspring with warm-acclimated parents, similar to the maximum metabolic rate findings of Donelson *et al*. (2012). When visualized as the response to an acute temperature increase (Fig. 1), we predicted an overall upward shift in the MO_2_-temperature relationship of offspring from warm-acclimated parents compared to those from cold-acclimated parents. Lastly, offspring with warm-acclimated parents would also have a higher CTM compared to those from cold-acclimated parents. Provided that resources are not limited, a higher peak MO_2_ and CTM would benefit the offspring overall, allowing them to tolerate warmer environments, though the trade-off would be an increased resting MO_2_ associated with a higher cost of living. If the hypothesis that the parents additively contribute to the transgenerational effect is correct, then the acclimation temperature of both parents will have an effect on the offspring’s response.

## Materials and methods

These experiments were conducted in accordance with the guidelines of the Canadian Council on Animal Care. They have been approved by the Institutional Animal Care Committee of Trent University (Protocol # 24794) and the Ontario Ministry of Natural Resources and Forestry (OMNRF) Aquatic Animal Care Committee (Protocol # 136).

The strain of lake trout used in this experiment originated from Seneca Lake, which is a glacial lake located in the Finger Lakes region in central New York State (42°41′ N, 76°54′ W). This strain has been kept in the OMNRF hatchery system for five generations since 1990 and has been maintained using rotational line crossing (Kincaid, 1977) to maintain its original genetic diversity and reduce inbreeding (OMNRF Fish Culture Stocks Catalogue, 2005).

### Experimental design: adult trout acclimation and breeding

Mature adult lake trout (age, 8; 2.3–4.2 kg) were held at the OMNRF White Lake Fish Culture Station (Sharbot Lake, Ontario, Canada) where they were individually PIT tagged (Oregon RFID, Portland OR), divided into two groups (*n* = 8 and 9, mixed sex) and acclimated to two different temperatures (10 ± 0.5°C and 17 ± 0.5°C) beginning in July 2015, by increasing temperatures 1°C per day until target temperatures were reached. The lower target temperature was based on lake trout temperature requirements for spawning and the elevated temperature was chosen to exceed their typical range but remaining within physiological limits (Casselman, 2008) with the aim of inducing a physiological stress response due to warming while attempting to avoid reproductive failure. Adults were housed in 1 × 1 × 6 m tanks that were covered with black tarpaulin to block out light. Temperatures were maintained by drawing water from above and below the thermocline in the hatchery’s water source (White Lake) and mixing it as it was fed into the tanks where the fish were held. After September, the temperature of each tank was allowed to follow the seasonal cooling of the lake.

Beginning in October, offspring were produced by dry-spawning anaesthetized fish (anaesthetic, 0.1 g l^−1^ MS-222; Aqua Life, Syndel Laboratories Ltd, BC, Canada) where 140 ml of eggs were stripped from each female, divided evenly among four jars and fertilized by pipetting milt directly onto them. Families were produced by a full factorial 4 × 4 mating cross using two males and two females from each of the two temperature treatments (eight fish in total) so that resultant offspring were from parents who had been acclimated to either the same or different temperatures prior to spawning. This resulted in four offspring families from each of the four parental treatment groups (W_♀_xW_♂_, W_♀_xC_♂_, C_♀_xW_♂_ and C_♀_xC_♂_, where W refers to a warm acclimated parent and C refers to a cold-acclimated parent) for a total of 16 families (Table S1). After fertilization, egg jars were kept cool and transported in coolers to the Codrington Fish Research Facility (Codrington, Ontario, Canada). Upon arrival, the eggs from each jar were placed in perforated steel boxes (9 × 9 × 7.5 cm, one family per box), which were kept in flow-through tanks receiving freshwater at ambient temperature (5–6°C) and natural photoperiod under dim light. To eliminate the potential effects of developmental plasticity with temperature on the metabolism of the offspring, we reared all eggs under the same temperature and lighting conditions.

### Experimental design: offspring temperature acclimation

In March, when the fry reached the exogenous feeding stage, 14 individuals from each family were randomly selected, split into two groups of seven and transferred into one of four larger (200 l) tanks. Each tank was separated into four sections to keep the families separate; however, due to space constraints, two families were kept in each tank section where the offspring from the two families sharing a section were half-siblings by their father. The individuals would later be identified to family using microsatellite genotyping (Supplemental File). Two tanks received a cold/optimal temperature (11°C) and the other two received a warm temperature (15°C) so that each family had seven representatives acclimated to each temperature. The lower acclimation temperature was selected based on the optimal growth temperature for lake trout (Edsall & Cleland, 2000; Casselman, 2008), and the warm temperature represents the potential warming in the Great Lakes region due to climate change by the end of the century (Hayhoe *et al*., 2010).

After transferring the fry to the larger tanks, we changed the water temperature at a rate of 1°C per day until the target temperatures (11 and 15°C) were reached, and the fish were acclimated for 3–4 weeks before the experiments began. The fish were fed 5–6 times a day at 2–3% their mass; however, fish were fasted for at least 12 hours prior to experimentation so that the physiological effects from recent feeding did not influence experimental results (Millidine *et al*., 2009).

### Respirometry set up

To test for potential transgenerational effects of the parental environment on offspring physiology, we measured and compared the metabolic rate of offspring of parents acclimated to matched or mismatched temperature conditions. To do so, we first measured the metabolic rate of offspring as the rate of oxygen consumption (MO_2_), using closed respirometry during an acute temperature increase (+2°C·h^−1^). From this dataset, we determined each individual offspring’s resting rate of oxygen consumption (MO_2_) and peak (thermally induced) MO_2_. The resting MO_2_ was recorded as the MO_2_ at the fish’s acclimation temperature before temperature began to rise with the acute temperature challenge, and the peak MO_2_ was recorded as the highest, thermally induced MO_2_ achieved during the trial. We distinguish peak MO_2_ from maximum MO_2_ (reported for exhaustive exercise protocols) because highest MO_2_ observed due to temperature may not necessarily represent the absolute maximum rate possible for each offspring. For this reason, we do not calculate aerobic scope. To determine the upper thermal tolerance in the offspring, we measured the CTM, which is the highest temperature that can be tolerated by the fish. This was recorded as the temperature at which the fish lost equilibrium as temperature increased, identified as a loss of dorsoventral orientation with the inability to right itself after 5–10 seconds.

Respirometers consisted of custom-built glass cylinders (8 cm diameter × 4.5 cm height, 226 cm^3^ volume) sealed at one end and fitted with an acrylic lid. Each lid had an inlet and outlet valve to allow water to flow through the chambers using a submersible pump that circulated water through the respirometers at 4.5 l min^−1^. The valves were situated on either side of a fitting that held a dissolved oxygen probe (model DO-BTA, Vernier Software and Technology, OR, USA) in place. The respirometers were contained in clear plastic tubs (two respirometers per tub) atop two side-by-side stir plates so that each respirometer was positioned over a stir plate. A magnetic stir bar in each respirometer was set to spin at ~60 RPM to keep water circulating in the chamber and a perforated stainless-steel grid separated the fish from the stir bar. The containers received aerated freshwater from a source tank that was temperature controlled using three 500 W titanium heaters (model TH-0500, Finnex, IL, USA) with digital temperature controllers (model HC 810 M, Finnex). The plastic tubs were covered in a sheet of thin, black plastic to minimize visual disturbance to the fish.

### Respirometry protocol and determining CTM

The night before the experiment, eight fish were individually transferred into separate respirometers where they received a continuous flow of fresh water maintained at their acclimation temperature and were left to adjust to the experimental apparatus overnight. Resting MO_2_ was measured the following morning. MO_2_ measurements were collected by manually switching off the pumps that circulated water through the respirometers and closing the input and output valves to create a closed system. The stir bar kept water moving past the oxygen probe which was connected to a Lab Pro (Vernier Software and Technology) interfaced with LoggerPro software (version 3.8.6; Vernier Software and Technology) so that the reduction in oxygen concentration could be recorded. Measurement of MO_2_ began after a 30 second wait period, then the drop in O_2_ was recorded for 10 minutes, after which the valves were opened to allow fresh water to flush the chamber until the next oxygen consumption measurement was made (~30 minutes). We observed the activity of the fish during each trial and an MO_2_ value was excluded from the analysis if a fish was active during the measurement period.

After measurement of resting MO_2_, fish were subjected to an acute temperature challenge of +2°C per hour by raising the temperature of the water in the source tank that fed the tubs housing the respirometers. We chose this rate to be consistent with previous studies that measured metabolic rate via oxygen consumption in related species (Penney *et al*., 2014). We measured MO_2_, for 10 minutes, at every 1°C increase until the fish lost equilibrium, which was observed when the fish could no longer maintain an upright position in the respirometer chamber, and this was recorded as the CTM for that fish. At this point, the focal fish was quickly removed from the chamber and euthanized in a bath of 0.3 g l^−1^ of tricaine methanesulfonate (MS-222; Aqua Life, Syndel Laboratories Ltd, BC, Canada). The focal fish was blotted dry on a paper towel so that mass (measured to the nearest 0.1 g using a digital balance scale) and fork length (measured to the nearest 1 mm using digital callipers) could be measured, and a caudal fin tissue sample (~0.25 cm^2^) was preserved in 95% ethanol for subsequent genotyping to identify offspring individuals to their respective family (see Supplementary Material).

The oxygen saturation of the water in the source tank and respirometers was continuously monitored throughout each trial. Oxygen saturation was 6.5–7.5 mg l^−1^ at the start of the measurement period; O_2_ saturation by the end of the measurement period varied depending on the temperature during the acute temperature challenge, where it ranged 4.5–5.5 mg l^−1^. The measurement period was shortened if oxygen concentration in the respirometers began to approach the critical limit of 3.5 mg O_2_ l^−1^ to attempt to minimize hypoxia-related responses in the fish (Doudoroff & Shumway, 1970; Cook *et al*., 2018). Also, if the oxygen saturation levels in the source tank began to drop due to higher temperatures, then oxygen was supplemented to the source tank water with a tank of compressed O_2_ and diffuser. Hyperoxia did not occur and O_2_ supplementation did not influence temperature during the experiment.

### Calculations and statistical analysis

Reduction in oxygen concentration was recorded as mg O_2_ l^−1^ min^−1^, and the rate of oxygen consumption (MO_2_) was calculated using the following formula:

$$ M{O}_2=\frac{\left( Rate\ of\ decline\ in\ \left[{O}_2\right]\right)\left({V}_R-{V}_F\right)\ x\ 60}{h} $$

where (Rate of decline in [O_2_]) is the decline in water oxygen concentration during the 10-minute measurement period, *V_R_* is the volume (l) of the respirometer, *V_F_* is the volume of the fish (l) and *h* is the time in hours.

Condition factor was calculated as follows:

$$ Condition\ factor=\frac{mass}{{\left( fork\ length\right)}^3}\ x\ 100. $$

To explore factors that contributed to variation in body mass and condition factor, we used JMP 13 (v. 18.1). Statistical analyses of the MO_2_ during the temperature challenge and the resting and peak MO_2_ were conducted using R (v. 3.5.2) with the ‘MuMIn’ (Barton, 2019), ‘lme4’ (Bates *et al*., 2015) and ‘mgcv’ (Wood, 2011) packages. The level of significance was set to 0.05 in all analyses, and all model assumptions (linearity, homogeneity of variance, sample independence and residual normality) were confirmed with Shapiro–Wilk W, Levene’s and Brown–Forsythe tests. In some cases, our response variable appeared non-normally distributed (according to Shapiro–Wilk W tests); however, we still opted for parametric tests as our selected analytical approaches are not highly sensitive to non-normality (Glass *et al*., 1972; Harwell *et al*., 1992; Lix *et al*., 1996; Bodden *et al*., 2017; Senduran *et al*., 2018) and depend more on homogeneity of variance instead. Lastly, we present MO_2_ using two terms: mass adjusted and mass specific. The mass-adjusted values are derived from the GLMM, which includes whole animal rates with mass as a covariate and are the values on which the statistical analysis was performed. The mass-specific values are the MO_2_ divided by mass and we include mass-specific values to present the MO_2_ data in a manner consistent and comparable with previous studies that include respirometry in fish.

The complexity of the experimental design (large number of fixed effects and interaction terms) and logistic limitations on sample size (number of independent crosses and available rearing space) prevented using conventional statistical analyses to assess the relative contributions of within and TGP. We tested a large number of fixed effects to determine how offspring upper thermal tolerance and metabolic rate changed with offspring acclimation temperature (within-generation plasticity), parental acclimation temperature (TGP) and their potential interactions. The list of fixed effects was further expanded by splitting parental acclimation temperature into maternal and paternal components to assess the relative parental contributions. Along with the interaction terms, this unavoidably gave rise to a complex global model.

To test for effects of maternal, paternal and offspring acclimation temperatures on offspring condition factor and mass, we used two separate general linear mixed effects models (GLMM) in JMP, with mass and condition factor as Gaussian-distributed response variables. These models both included offspring acclimation temperature (cold or warm) and parent acclimation temperature as fixed effect predictors, where parents were treated as a single explanatory variable with mother and father acclimation temperature combined and represented as one of four fixed effects: C_♀_xC_♂_, C_♀_xW_♂_, W_♀_xC_♂_ or W_♀_xW_♂_ (C = cold acclimation and W = warm acclimation). To test for whether parental acclimation temperature yielded differential effects on mass and condition of offspring reared in cold or warm water, an interaction between offspring and parental treatment group was also included as a fixed effect predictor. Degree days was included as a random intercept to control for effects of age on mass and condition, since the experiment lasted ~5 weeks and most of the cold-acclimated offspring were tested in the first half of the experimental period. Here, degree days were calculated for each fish as the cumulative temperature experienced above 0°C (Chezik *et al*., 2013; Cook *et al*., 2018) until the day of the experiment. Finally, offspring identity (ID) and parental IDs (ID*_M_* and ID*_F_*) were also included as random intercepts to account for statistical non-independence between offspring that were sired or dammed from the same parents.

To test the effect of maternal, paternal and offspring acclimation temperature on the metabolic (MO_2_) response of the offspring to an acute temperature challenge, we again used a GLMM, using the ‘nlme’ package in R (Pinheiro *et al*., 2019) to permit correction for temporal autocorrelation. In this model, MO_2_ was used as a Gaussian-distributed response variable, with acute challenge temperature (*T_a_*; continuous variable), offspring acclimation temperature (*T_O_*; cold and warm) and acclimation temperatures of the mothers (*T_M_*; cold and warm) and fathers (*T_F_*; cold and warm) as fixed effect predictors, along with all possible interactions between these terms. Additionally, *Mass* (fixed term) was included in the model as a continuous predictor because metabolic rate scales with mass and the warm-acclimated offspring grew heavier than cold-acclimated ones. Similar to our previous models, both mother and father ID (ID*_M_* and ID*_F_*) were included as random intercepts to account for relatedness among offspring, and offspring ID was included as a random intercept to control for statistical non-independence between measurements drawn from the same individual.

Because the relationship between MO_2_ and acute temperature challenge was curvilinear and could not be predicted by a simple polynomial function (i.e. with relatively low degree), we first modelled the relationship between MO_2_ and acute challenge temperature alone using a cubic regression spline in a general additive model (GAM), using three knots to appropriately capture the shape of the relationship while avoiding over-fitting. Predicted MO_2_ at each challenge temperature was extracted for each offspring and used in place of acute challenge temperature within our GLMM to account for the variation in the response variable (observed MO_2_) due to acute challenge temperature. This approach permitted us to (i) remove the complex, curvilinear relationship between MO_2_ and acute challenge temperature, (ii) test whether the remaining variation in MO_2_ (i.e. that not explained by acute challenge temperature) can be explained by the other terms in the GLMM and (iii) include multi-level interactions between a previously non-linear predictor (acute challenge temperature) and additional factorial predictors, which cannot be accomplished simply with current additive models. Finally, to account for heterogeneity of variance in MO_2_ across acute challenge temperature (and detected across predicted MO_2_) and to correct for autocorrelation between measurements drawn at adjacent time-points, we weighted our model by acute challenge temperature and included a type I autoregressive correlation structure, with an estimated ρ of 0.397.

An effect of parental acclimation temperature on the resting MO_2_, peak MO_2_ and CTM of offspring was analyzed using three independent linear mixed models in R, each with the ‘lme4’ package (Bates *et al*., 2015). Here, we first sought to include mother acclimation temperature (*T_M_*), father acclimation temperature (*T_F_*) and offspring acclimation temperature (*T_O_*) as fixed effect predictors, with all possible interactions between each of these factors, along with offspring *mass* as a covariate. Unfortunately, however, our total number of observations per experimental group ($\overline{n}$ = 21.125 ± s.d. = 2.642; total *n* = 157) was too few to support a robust approach such as an analysis of variance to test the individual effects of each predictor (π = 0.448; for expected relationships with weak explanatory capacity; Cohen’s *f*^2^  $\cong$ 0.05; as tested using the ‘pwr’ package in r; Champely *et al*., 2018). We therefore used an Akaike information criterion (AIC) approach to identify which models best explained the variation in the data while avoiding over-parameterization, using the previously described global model, including mother ID (ID*_M_*) and father ID (ID*_F_*) as random intercepts to account for relatedness among offspring. The best models were considered as those with a ΔAIC ≤ 2 as recommended by Burnham and Anderson (2002) where the ΔAIC was calculated as the difference in the AIC value of a given model versus the top model (i.e. the model with the lowest AIC value). We also calculated the evidence ratio (ER) and Akaike weight (Wi) of each model iteration. The ER is the likelihood that the top model is the best supporting model compared to another model, and the Wi is the weight (or proportion) of evidence that a given model best explains the variation in the data (Burnham & Anderson, 2002). These metrics were used to compare the best models and to observe common parameters among these models.

## Results

### Mass and condition factor

On average, warm-acclimated offspring were nearly twice as heavy compared with cold-acclimated offspring (least squares means, 4.28 ± 0.14 g versus 2.76 ± 0.13 g; GLMM: *F*_1,21.28_ = 93.58, *P* < 0.001; Table 1). Offspring mass did not differ among parental groupings (C_♀_xC_♂_, C_♀_xW_♂_, W_♀_xC_♂_, W_♀_xW_♂_) (GLMM: *F*_3,3.63_ = 1.08, *P* = 0.45), but there was an interaction between offspring acclimation and parental group (GLMM: *F*_3,46.53_ = 4.23, *P* = 0.01).

**Table 1:** The mass and condition factor of 11 and 15°C acclimated lake trout offspring

	11°C Acclimated offspring				15°C Acclimated offspring			
Parental group	C_♀_xC_♂_	C_♀_xW_♂_	W_♀_xC_♂_	W_♀_xW_♂_	C_♀_xC_♂_	C_♀_xW_♂_	W_♀_xC_♂_	W_♀_xW_♂_
Mass (g)	2.93 (±0.26)	2.69 (±0.23)	3.26 (±0.22)	2.14 (±0.26)	4.16* (±0.25)	4.61* (±0.28)	4.17* (±0.25)	4.19* (±0.27)
Condition factor	0.91 (±0.01)	0.88 (±0.01)	0.91 (±0.01)	0.86 (±0.01)	0.93 (±0.01)	0.94 (±0.01)	0.94 (±0.01)	0.92 (±0.01)

Parental groups are represented as the maternal environment crossed with the paternal environment: C♀xC♂, C♀xW♂, W♀xC♂ and W♀xW♂ where C = cold and W = warm. Values are least squares means ± SEM. Statistical significance (P < 0.05; GLMM) between offspring acclimation temperature is indicated by an asterisk.

**Table 2:** Summary of the GLMM results to test for a transgenerational effect of acclimation temperature on lake trout offspring MO_2_ during an acute temperature challenge

Parameter	Coefficient	S. E.	DF	*t*-value	*P*-value
Intercept	−0.32	0.07	18.45	−4.60	**<0.001**
Mass	0.14	0.01	155.32	10.66	**<0.001**
T_a_	0.89	0.05	2212.78	17.58	**<0.001**
T_O_	−0.30	0.09	1023.18	−3.40	**<0.001**
T_M_	0.17	0.08	7.73	2.12	0.068
T_F_	0.08	0.07	560.72	1.22	0.222
T_O_ · T_M_	−0.20	0.12	1158.94	−1.66	0.097
T_O_ · T_F_	−0.44	0.13	1247.21	−3.42	**<0.001**
T_F_ · T_M_	0.09	0.09	501.51	1.01	0.312
T_a_ · T_O_	0.24	0.09	2207.42	2.61	**0.009**
T_a_ · T_M_	−0.29	0.07	2209.73	−4.34	**<0.001**
T_a_ · T_F_	−0.23	0.07	2209.80	−3.39	**<0.001**
T_O_ · T_M_ · T_F_	0.16	0.18	1233.62	0.92	0.360
T_a_ · T_O_ · T_M_	0.30	0.13	2206.66	2.34	**0.019**
T_a_ · T_O_ · T_F_	0.62	0.14	2210.05	4.46	**<0.001**
T_a_ · T_M_ · T_F_	0.04	0.10	2207.83	0.46	0.641
T_a_ · T_O_ · T_M_ · T_F_	−0.30	0.19	2208.18	−1.60	0.109

Offspring were from parents acclimated to either a cold or warm temperature. The offspring were also acclimated to cold or warm temperature. *T_F_*, *T_M_* and *T_O_* are the father, mother and offspring acclimation temperatures, respectively, and the acute temperature challenge is represented by *T_a_*. Significant effects (*P* < 0.05) are highlighted with bold text.

Warm-acclimated offspring had significantly higher body condition than cold-acclimated offspring with means of 0.93 ± 0.01 versus 0.89 ± 0.01 (Table 1), respectively (GLMM: *F*_1,20.47_ = 38.67, *P* < 0.001). Offspring condition factor was not affected by parent acclimation temperatures (GLMM: *F*_3,3.23_ = 1.83, *P* = 0.31) nor by the interaction between offspring acclimation and parental group (GLMM: *F*_3,2.07_, *P* = 0.12).

### Offspring metabolic rate with an acute temperature increase

For the effect of the acute temperature challenge on offspring metabolic rate, there was an increase in offspring mass-adjusted MO_2_ with increasing body mass (*Mass*: *t* = 10.66, *P* < 0.001, Table 2). Offspring MO_2_ also increased with challenge temperature (GAMM: *T_a_*: *t* = 17.58, *P* < 0.001). Offspring acclimation temperature (*T_O_*) had a significant effect on MO_2_ with warm-acclimated offspring having a higher MO_2_ (*T_O_*: *t* = −3.40, *P* < 0.001, Table 2). Neither maternal nor paternal acclimation temperature in isolation was strong enough to influence offspring MO_2_ (*T_M_*: *t* = 2.12, *P* = 0.068; *T_F_*: *t* = 1.22, *P* = 0.222; Table 2).

While the interaction between offspring and maternal acclimation temperature was not significant (*T_O_ · T_M_*: *t* = −1.66, *P* = 0.097), the interaction between offspring and paternal acclimation temperature did influence MO_2_ (*T_O_ · T_F_*: *t* = −3.42, *P* < 0.001). There was no significant interaction between mother and father acclimation temperature on the offspring’s metabolic response (*T_M_ · T_F_*: t = 1.01, p = 0.312). Significant two-way interactions occurred between *T_a_* and *T_O_* (*t* = 2.61, *P* = 0.009) demonstrating that some remaining variation in MO_2_ that was not explained by challenge temperature could be explained by offspring acclimation temperature; more specifically, offspring reared at warm temperatures appeared to respond differently to the thermal challenges than did cold-acclimated offspring. The acclimation temperature of the parents interacted significantly with the acute temperature challenge to determine the offspring’s MO_2_ (*T_a_* · *T_M_*: *t* = −4.34, *P* < 0.001; *T_a_* · *T_F_*: *t* = −3.39, *P* < 0.001; Table 2). The metabolic response of the offspring also depended on the complex interaction between offspring acclimation temperature, parental (maternal or paternal) acclimation temperature and challenge temperature (*T_a_ · T_O_ · T_M_*, *t* = 2.34, *P* = 0.019; *T_a_ · T_O_ · T_F_*, *t* = 4.46, *P* < 0.001; Table 1). No other main effect interactions were significant (Table 2).

**Figure 2:**
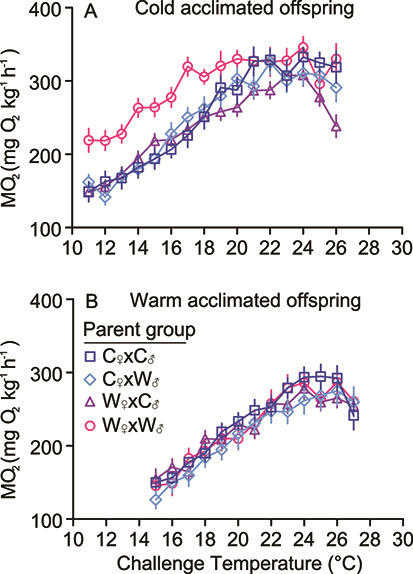
The change in the rate of oxygen consumption (MO_2_) of (**A**) cold and (**B**) warm acclimated lake trout offspring given an acute temperature challenge of +2°C·h^−1^, showing mass-specific means ± SEM. Parental groups are represented as the maternal environment crossed with the paternal environment: C_♀_xC_♂_, C_♀_xW_♂_, W_♀_xC_♂_ and W_♀_xW_♂_ where C = cold and W = warm.

To visually explore maternal and paternal influences on offspring MO_2_, we plotted the mass-specific MO_2_ for offspring from different parental combinations against the acute temperature challenge (Fig. 2). We did not perform a statistical analysis on the mass-specific values because the GLMM (previously described) tested MO_2_ while accounting for mass in the model. Qualitatively, the cold-acclimated offspring from warm-acclimated parents (W_♀_xW_♂_) had a higher metabolic rate at the beginning of the temperature challenge compared with the other parental acclimation groups (C_♀_xC_♂_, C_♀_xW_♂_, W_♀_xC_♂_; Fig. 2A) indicating that an environmental mismatch between generations can influence offspring metabolic response. This effect did not carry over to the warm-acclimated offspring (Fig. 2B) as MO_2_ was comparable among the parental acclimation groups.

**Figure 3:**
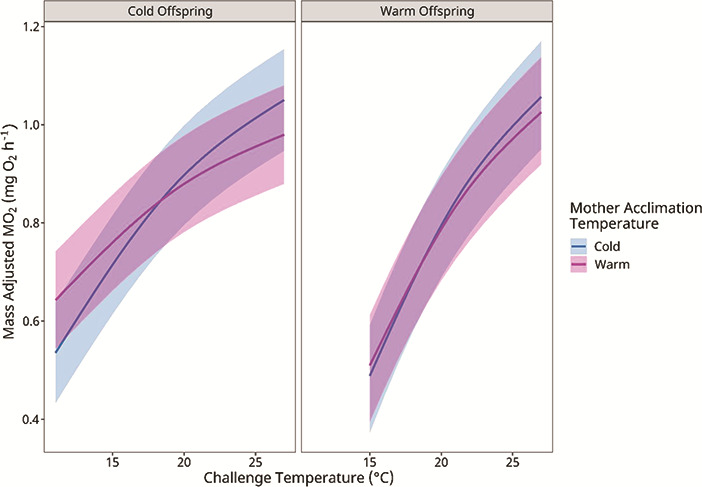
The influence of maternal acclimation temperature on the change in the rate of oxygen consumption (MO_2_) of cold and warm acclimated lake trout offspring given an acute temperature challenge of +2°C·h^−1^. Values are means estimated from the GLMM with 95% confidence intervals (refer to Methods).

To visually isolate the effects of maternal acclimation temperature on offspring’s thermal response, we plotted the mass-adjusted MO_2_ (Fig. 3) estimated from the GLMM to show the interaction between challenge temperature and the acclimation temperature of the mothers (*T_a_ · T_M_*; Table 2). For both cold- and warm-acclimated offspring (Fig. 3, both panels), the difference in the slope of the MO_2_-temperature relationship illustrates the significant interaction between challenge temperature and acclimation temperatures of the offspring and mothers (*T_a_ · T_O_ · T_M_*; *P* = 0.019; Table 2). Focusing on the cold-acclimated offspring (Fig. 3, left), at cooler challenge temperatures, the MO_2_ of offspring from warm-acclimated mothers was elevated compared to offspring from cold-acclimated mothers. For warm-acclimated offspring at challenge temperatures below ~19°C, individuals from warm-acclimated mothers had a higher MO_2_ compared to those from cold-acclimated mothers (Fig. 3, left). This general trend occurred to a lesser extent in the warm-acclimated offspring (Fig. 3, right) with the lines of the offspring’s MO_2_-temperature relationship overlapping for warm- and cold-acclimated mothers.

**Figure 4:**
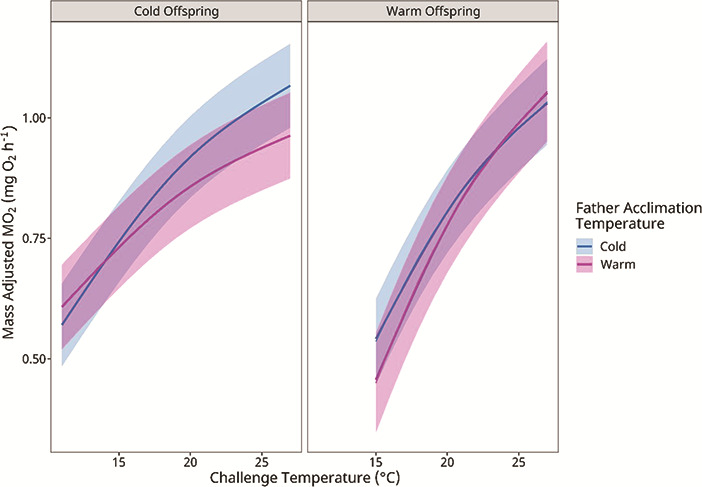
The influence of paternal acclimation temperature on the change in the rate of oxygen consumption (MO_2_) of cold and warm acclimated lake trout offspring given an acute temperature challenge of +2°C·h^−1^. Values are means estimated from the GLMM with 95% confidence intervals (refer to Methods).

To visually explore the paternal effect on offspring MO_2_, we plotted the mass-adjusted MO_2_, estimated from the GLMM (Fig. 4). This illustrates the significant interaction between challenge temperature and the acclimation temperatures of the offspring and fathers (*T_a_ · T_O_ · T_F_*; *P* < 0.001; Table 2). For cold-acclimated offspring, the MO_2_-temperature relationship of those from warm-acclimated fathers was above that of those from cold-acclimated fathers with the lines crossing at ~14°C (Fig. 4, left). A reverse trend occurred in the warm-acclimated offspring as individuals from warm-acclimated fathers had a lower MO_2_ at cooler challenge temperatures compared to those from cold-acclimated fathers (Fig. 4, left).

### Resting and peak metabolic rates

An analysis of resting MO_2_ with AIC revealed six models (ΔAIC ≤ 2) that best predicted the trends in the data with *Mass* appearing in each of these models. The first model contained *Mass* as the only fixed variable, but this model was only 1.23 times more likely (evidence ratio, ER) than model 2, which included maternal (*T_M_*) and paternal (*T_F_*) acclimation temperature with their interaction, to best explain the variation in the data (Table 3). Interestingly, offspring acclimation temperature (*T_O_*) appeared only twice among the six models. Maternal (*T_M_*) and paternal (*T_F_*) acclimation temperature appeared most frequently among the six models, with an interaction between these terms appearing in two of these. An interaction between offspring and paternal acclimation temperature (*T_O_ · T_F_*) appeared in only one of the models (Table 3). Altogether, this suggests that maternal and paternal environments, individually and combined, can act on the response of the offspring’s resting metabolic rate to temperature acclimation.

**Table 3:** Summary of the top models determined with AIC to explain variation in resting MO_2_, peak (highest achieved, thermally induced) MO_2_ and CTM with transgenerational acclimation of lake trout offspring

Measure	Model #	ΔAIC	ER	*W_i_*	*R* ^2^	Model
Resting MO_2_	1	0	1.00	0.25	0.41	Mass + ID_M_ + ID_F_
	2	0.42	1.23	0.20	0.43	Mass + *T_F_* + *T_M_* + (*T_F_* · *T_M_*) + ID*_M_* + ID*_F_*
	3	0.58	1.34	0.18	0.45	Mass + *T_F_* + *T_M_* + *T_O_* + (*T_F_* · *T_M_*) + (*T_F_* · *T_O_*) + ID*_M_* + ID*_F_*
	4	1.16	1.79	0.13	0.42	Mass + *T_M_* + ID*_M_* + ID*_F_*
	5	1.53	2.14	0.12	0.42	Mass + *T_F_* + ID*_M_* + ID*_F_*
	6	1.55	2.17	0.11	0.42	Mass + *T_O_* + ID*_M_* + ID*_F_*
Peak MO_2_	1	0	1.00	0.33	0.64	Mass + ID*_M_* + ID*_F_*
	2	0.77	1.47	0.23	0.65	Mass + *T_F_* + *T_M_* + (*T_F_* · *T_M_*) + ID*_M_* + ID*_F_*
	3	1.24	1.86	0.18	0.64	Mass + *T_M_* + ID*_M_* + ID*_F_*
	4	1.73	2.38	0.14	0.64	Mass + *T_F_* + ID*_M_* + ID*_F_*
	5	1.97	2.68	0.12	0.64	Mass + *T_O_* + ID*_M_* + ID*_F_*
CTM	1	0	1.00	0.39	0.29	Mass + *T_F_* + *T_M_* + *T_O_* + (*T_F_* · *T_M_*) + (*T_F_* · *T_O_*) + (*T_M_* · *T_O_*) + (*T_F_* · *T_M_* · *T_O_*) + ID*_M_* + ID*_F_*
	2	0.67	1.40	0.28	0.27	*T_F_* + *T_M_* + *T_O_* + (*T_F_* · *T_M_*) + (*T_F_* · *T_O_*) + (*T_M_* · *T_O_*) + (*T_F_* · *T_M_* · *T_O_*) + *ID_M_* + ID*_F_*
	3	1.65	2.28	0.17	0.27	Mass + *T_O_* + ID*_M_* + ID*_F_*
	4	1.87	2.55	0.15	0.25	*T_O_* + ID*_M_* + ID*_F_*

Offspring were from parents acclimated to either a cold or warm temperature and were also acclimated to cold or warm temperature. *T_F_*, *T_M_* and *T_O_* are the father, mother and offspring acclimation temperatures, respectively, and *ID_M_* and *ID_F_* are the mother and father individual identification (treated as random effects), which appear in each model to account for offspring relatedness.

**Figure 5:**
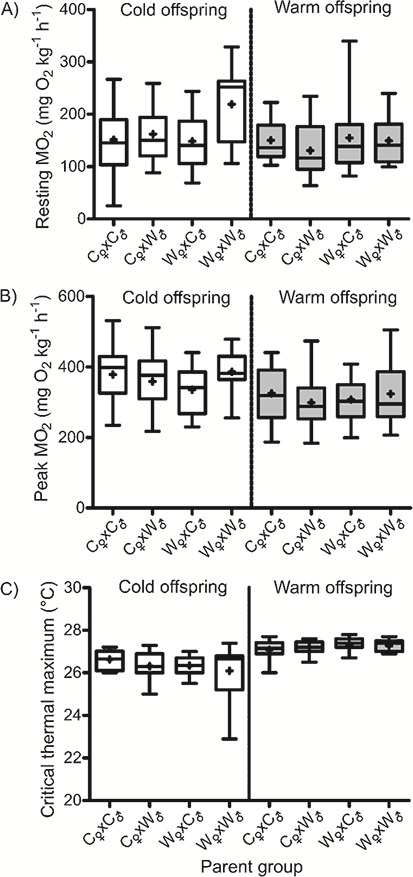
(**A**) Resting rate of oxygen consumption (MO_2_), (**B**) peak (highest achieved, thermally induced) MO_2_ and (**C**) CTM of lake trout offspring acclimated to a cold (open) or warm (shaded) temperature. Parental groups are represented as the maternal environment crossed with the paternal environment: C_♀_xC_♂_, C_♀_xW_♂_, W_♀_xC_♂_ and W_♀_xW_♂_ where C = cold and W = warm. The plot shows the 25th and 75th quartiles with medians; means are represented as ‘+’, and the upper and lower tails are the minimum and maximum values.

We plotted the resting MO_2_ (mass-specific values, no statistical analysis) to visually explore trends within and between the offspring and parental acclimation groups (Fig. 5A). The cold-acclimated offspring from warm-acclimated parents (open boxes, W_♀_xW_♂_, Fig. 5) had the highest mean resting MO_2_ (219.2 ± 15.97 mg O_2_ kg^−1^ h^−1^), while the resting MO_2_ of the other three groups ranged between 148.3 ± 11.08 and 162.1 ± 10.51 mg O_2_ kg^−1^ h^−1^ (Fig. 5A). There was no observable trend for warm-acclimated offspring (shaded boxes, Fig. 5A) as resting MO_2_, irrespective of parental acclimation temperature, ranged between 130.4 ± 12.56 and 154.7 ± 13.52 mg O_2_ kg^−1^ h^−1^. When comparing offspring within parental acclimation temperatures (open versus shaded boxes; Fig. 5A), the mean resting MO_2_ of cold-acclimated offspring from warm-acclimated parents (shaded boxes, W_♀_xW_♂_; Fig. 5A) was higher compared to cold-acclimated offspring (open boxes, W_♀_xW_♂_; Fig. 5A).

We also used an information theoretic approach to explore factors contributing to variation in peak MO_2_. Of the five models that best explained the variation in peak MO_2_, *mass* appeared in each model with the top model (ER = 1, *W_i_* = 0.33) containing *mass* as the only fixed parameter (Table 3). Maternal (*T_M_*) and paternal (*T_F_*) acclimation temperature, and the interaction term between the two, occurred in the second-best model, which had a 23% (*W_i_*) chance of being the top model (Table 3). The other three of the five models contained only one fixed parameter: either *T_O_*, *T_M_* or *T_F_* (Table 3).

Peak MO_2_ (mass-specific values, no statistical analysis) was also plotted to visually explore trends within and between the offspring and parental acclimation groups (Fig. 5B). Overall, cold-acclimated offspring attained a higher mean peak MO_2_ (mass-specific) than warm-acclimated offspring (open versus shaded boxes, W_♀_xW_♂_, C_♀_xW_♂_, W_♀_xC_♂_, C_♀_xC_♂_; Fig. 5B). When comparing offspring within an acclimation temperature, peak MO_2_ was comparable; cold-acclimated offspring (open boxes, Fig. 5B) ranged between 335.8 ± 12.97 and 386.6 ± 13.13 mg O_2_ kg^−1^ h^−1^, and warm-acclimated offspring (shaded boxes, Fig. 5B) ranged between 299.2 ± 18.79 and 325.5 ± 16.02 mg O_2_ kg^−1^ h^−1^.

### Critical thermal maximum

Four AIC models best explained the trends in the CTM data (ΔAIC ≤ 2). Models 1 and 2 together suggest that CTM depended on a complex interaction between offspring (*T_O_*) and parental acclimation temperature (*T_M_* and *T_F_*). The top model (*W_i_* = 0.39) was the global model containing *Mass* as a covariate with the offspring (*T_O_*), maternal (*T_M_*) and paternal (*T_F_*) acclimation temperature as fixed effects and all two-way and three-way interaction terms between them (Table 3). The second model was also the global model excluding *Mass*; however, model 1 was 1.40 (ER) times more likely to explain variation in CTM compared to model 2 (Table 3). The third model contained only *Mass* and offspring acclimation temperature (*T_O_*), whereas as the fourth model contained only *T_O_* (Table 3).

CTM within and between the groups of offspring showed subtle differences (Fig. 5C). The mean CTM was comparable among groups of cold-acclimated offspring (open boxes, Fig. 5C) with values ranging between 26.10 ± 0.2 and 26.64 ± 0.10°C. Likewise, the CTM of warm-acclimated offspring (shaded boxes, Fig. 5C) was similar. When comparing offspring within parental acclimation groups, warm-acclimated offspring (shaded boxes) from W_♀_xW_♂_ parents had a CTM 1.17°C higher than that of cold-acclimated offspring (open boxes) from the same parental group (W_♀_xW_♂_, Fig. 5C). For the rest of the parental groups (C_♀_xW_♂_, W_♀_xC_♂_, C_♀_xC_♂_;), CTM was comparable (open vs. shaded boxes, Fig. 5C).

## Discussion

Lake trout families exhibited evidence of limited TGP, although the effects of TGP on MO_2_ and CTM were minor compared with offspring mass and acclimation temperature. Although warm-acclimation of the parents did not shift their offspring’s MO_2_-temperature relationship upward as predicted, we found that offspring thermal performance depended on complex interactions between parent and offspring environments. Ours is one of the few studies to investigate the relative parental contribution to TGP in a vertebrate offspring’s phenotype (Shama *et al*., 2014; Hellmann *et al*. 2020), and we demonstrate that the parents additively contribute to the limited TGP we observed (resting and peak MO_2_ and CTM).

Offspring MO_2_ was most strongly influenced by mass and acclimation temperature. This is not surprising given that warm-acclimated offspring were heavier than their cold-acclimated siblings and thus had higher whole-animal O_2_ consumption rates. The effect of allometric scaling was apparent when the MO_2_ was expressed mass specifically, where mean MO_2_ was higher in cold-acclimated offspring overall compared with warm-acclimated offspring (Clarke & Johnston, 1999). Although it is possible that partial hypoxia may have influenced offspring performance, we saw no evidence for this. The observed effect of offspring acclimation temperature on MO_2_ and CTM concurs with other lake trout studies, including evidence of limited acclimation capacity (Evans, 2007; McDermid *et al.*, 2013; Kelly *et al*., 2014). Lastly, individual (random) effects had a significant contribution but epigenetic priming can be expected to vary within and among individuals; thus, the extent of TGP should be expected to vary among both adults and offspring.

### Limited evidence for TGP

At lower challenge temperatures (11–20°C), the average MO_2_ of cold-acclimated offspring from warm-acclimated parents was elevated compared to those from cold-acclimated parents (Fig. 2A), suggesting a higher cost of living (Norin & Metcalfe, 2019) at these temperatures when an environmental mismatch exists between generations. At warmer acute challenge temperatures (>18°C), cold-acclimated offspring from the different mating crosses showed similar MO_2_ values, and MO_2_ began to decline once temperature exceeded 24°C (Fig. 2A). This disagrees with previous findings that warm-acclimated offspring from warm-acclimated parents had a lower metabolic rate (Donelson *et al*., 2012), although the effect of TGP can be difficult to predict and may not always be to the benefit of the offspring (Guillaume *et al*., 2016). In addition to the acute temperature challenge, the resting MO_2_ of offspring from warm-acclimated parents (a generational environmental mismatch) was the highest among of the cold-acclimated offspring (Fig. 5A), and parent acclimation temperature did not have an appreciable effect on peak MO_2_ (Fig. 5B). While this contrasted with our predictions, standard metabolic rate is thought to be relatively plastic (Norin & Metcalfe, 2019), which may explain why resting MO_2_ was elevated in cold-acclimated offspring from warm acclimated parents in our study. We interpret the high resting MO_2_ in this group (11°C acclimated offspring from warm acclimated parents) to be due to an increased effort into surviving elevated temperature. It was evident that this group was not diverting energy into increased growth considering that the mass and condition factor were no greater in this group compared to the others.

MO_2_-temperature relationships were similar for the warm-acclimated offspring regardless of parental treatment (Fig. 2B), suggesting that parental environmental temperatures had little influence on offspring performance. This was surprising given that previous studies on fish have reported that offspring from warm-acclimated parents could tolerate warm temperatures better than offspring from cold-acclimated parents by reducing standard metabolic rate or increasing maximum metabolic rate (Donelson *et al*., 2012; Shama *et al*., 2014; Donelson *et al*., 2018). These earlier studies tested TGP in tropical or eurythermal species; thus, it is possible that TGP is limited in stenothermal species like lake trout. Limited TGP may also be related to the limited variation in within-generation thermal plasticity in lake trout (Evans, 2007; Kelly *et al*., 2014), and it could be that lake trout simply do not have the capacity to extend their thermal tolerance (Evans, 2007; McDermid *et al.*, 2013; Kelly *et al*., 2014). It is possible that multiple generations of exposure to the same stressor may be required to strengthen the effect (Burggren, 2015; Bell & Hellmann, 2019; Pilakouta *et al*., 2020), as in the case of the polychaete, *Ophryotrocha labronica*, where the effect of multigenerational exposure to warming was strongest in the F5 and F6 generations (Gibbin *et al*., 2017).

Our results showed that the thermal experiences of the parents had a relatively minor role in shaping the metabolic rate of the next generation in lake trout. Although we did not explore the physiological mechanisms underlying variation in offspring metabolic rate, TGP has been shown in other species to act on physiological mechanisms that can affect metabolic rate, such as mitochondrial function and gene expression (Shama *et al*., 2014; Gibbin *et al*., 2017). For example, mitochondria from the heart tissue of warm-acclimated stickleback offspring from warm-acclimated mothers had a lower rate of oxidative phosphorylation and less proton leak at warm temperatures than those from mothers acclimated to a cooler temperature, suggesting that offspring mitochondrial function is more efficient when maternal and offspring environments match (Shama *et al*., 2014). TGP has also been shown to up- or down-regulate the expression of genes involved in the heat shock response, metabolism, protein catabolism, immune response and reproduction (Veilleux *et al*., 2015; Shama *et al*., 2016; Veilleux *et al*., 2018).

### Additive parental contribution

Our results suggest that the contribution of the parents to TGP was additive. The offspring’s overall MO_2_ response to the acute temperature challenge was not influenced by the sole effect of either the maternal or paternal acclimation temperature, but instead on the complex interaction of maternal or paternal acclimation temperature with challenge temperature and offspring acclimation temperature (Table 2). The additive effect of parental temperatures on offspring metabolic rate was confirmed when both paternal and maternal acclimation temperature appeared in the top AIC models for resting and peak MO_2_ (Table 3). Both parents also contributed to their offspring’s upper thermal tolerance (CTM; Table 3) even though the differences in CTM among the groups of offspring were very slight (Fig. 5C). Similarly, Massamba-N’Siala *et al*. (2014) found no effect of TGP on the upper thermal tolerance of polychaetes given an acute temperature challenge.

TGP can be mediated through epigenetic modifications (summarized by Donkin & Barrès, 2018) that can be transmitted to the next generation (Crean & Bonduriansky, 2014; Marshall, 2015), but other non-genetic effects (maternal and paternal) could have influenced the offspring’s phenotype (Burgess & Marshall, 2011; Shama, 2015). Females, for example, can provision their eggs through changes in egg size or nutrient enrichment of the yolk, which can contribute to offspring fitness (Einum & Fleming, 1999; Gagliano & McCormick, 2007; Jonsson & Jonsson, 2016). We conducted a preliminary analysis of egg size, mass and water and energy content but did not find evidence of maternal provisioning relating to the trends observed in MO_2_ (Table S2). Similarly, Shama *et al*. (2014) found that temperature acclimation of the parents did not affect egg size. Another maternal effect includes the transfer of hormones, such as thyroid or cortisol, to the eggs, which could potentially alter offspring gene expression (Sopinka *et al*., 2017), growth and development (Gagliano & McCormick, 2009; Ruuskanen & Hsu, 2018). While we did not test the hormone content of the eggs, we acknowledge that it could potentially influence metabolic rate (Burton *et al*., 2011) of the offspring in our study.

The paternal contribution to TGP is understudied relative to maternal effects, although there is some evidence for non-genetic paternal effects (Crean & Bonduriansky, 2014; Marshall, 2015; Immler, 2018). The contribution of the father’s thermal environment to the offspring’s phenotype is variable, with effects seen in some species (e.g. marine tubeworm, Guillaume *et al*., 2016) but not others (e.g. stickleback, Shama *et al*., 2014). Further, paternal contributions to TGP can extend beyond the transmission of epigenetic machinery to their offspring, as ejaculate and sperm cytoplasmic components can also mediate paternal effects (Crean & Bonduriansky, 2014; Kekäläinen *et al*., 2018). How or if these components could have affected the metabolic response of the offspring to temperature stress in our study was not assessed.

### Perspectives and future directions

The importance of TGP may be a function of generation time and environmental fluctuation where TGP would be beneficial when the environment fluctuates predictably over multiple generations (Yin *et al*., 2019). Lake trout have a long generation time for a freshwater fish, reaching maturity in ~ 6–12 years depending on latitude and lake productivity (Martin & Olver, 1980; Hansen *et al*., 2012), and occupy thermally stable habitats with limited seasonal variation (Wilson & Mandrak, 2004; Guzzo & Blanchfield, 2017). Under these circumstances, TGP is unlikely to provide an ecologically significant benefit to lake trout populations. It is also important to note that an evolutionary response would require multiple generations and be dependent on existing heritable variation within local populations, which is likely limited in lake trout (Wilson & Mandrak, 2004; Perrier *et al*., 2017). Adaptation would require diversity at multiple genes involved in core metabolic pathways with adaptive responses acting in concert (Willi *et al*., 2006). Within- and among-population phenotypic variation in upper thermal tolerance is limited in lake trout (McDermid *et al*., 2013; Kelly *et al*., 2014) suggesting that standing genetic variation for genes underlying their thermal physiology is likely very limited.

Although TGP may have an important role in adaptation (Bernatchez, 2016; Smith *et al*., 2016), cold-adapted species with long generation times may not be able to keep up with the pace of anthropogenic climate change (Willi *et al*., 2006; Munday *et al*., 2013; Wilson *et al*., 2014). Based on our findings, it is unlikely that TGP effects in lake trout would be enough to sufficiently mitigate climate-related selection pressures to make much difference for population persistence under rapidly changing environmental conditions. Lake trout retreat to the cooler hypolimnion during the warmer summer months when the lake thermally stratifies (Casselman, 2008; Guzzo & Blanchfield, 2017), but climate change is expected to increase lake surface temperatures and prolong the duration of stratification (Lehman, 2002). For this reason, lake trout may be forced to reside in the hypolimnion for an extended period, lengthening their exposure to hypoxia, which could negatively impact important life history traits (Evans, 2007; Guzzo & Blanchfield, 2017).

From other studies, it is evident that TGP has some role to play in ‘priming’ offspring’s response to elevated temperatures (Yin *et al*., 2019; but see Sánchez-Tójar *et al*., 2020); however, TGP had only a limited effect on lake trout thermal performance in our study. A further investigation into how TGP acts to influence physiological processes is warranted and will require examination of the mechanisms underlying thermal tolerance, such as mitochondrial performance and gene expression in tandem with investigating which parental effects, including epigenetic inheritance (e.g. methylation, RNA interference), contribute to TGP. An understanding of how phenotypic plasticity, developmental plasticity, TGP and genetic changes combine to influence the adaptation of populations to climate change will not only help us anticipate the effects of a changing environment but will also deepen our knowledge of the link between plasticity, acclimation and adaptation.

## Authors’ contributions

CMP, GB and CCW conceived the ideas and designed methodology; CMP collected the data; CMP and JR analysed the data; CMP led the writing of the manuscript. All authors contributed critically to manuscript drafts and gave final approval for publication.

## Funding

This work was supported by Canada-Ontario Agreement on Great Lakes Water Quality and Ecosystem Health (COA) funding to CCW.

## Supplementary Material

ConservPhysiol_SupplementaryInfo_Final_clean_coab021
