# Supplementary material for: Limited transgenerational effects of environmental temperatures on thermal performance of a cold-adapted salmonid
Source: Conserv Physiol. 2021 Apr 24;9(1):coab021. doi: 10.1093/conphys/coab021 (PMC8071478; doi:10.1093/conphys/coab021)
Supplement: ConservPhysiol_SupplementaryInfo_Final_clean_coab021 [file conservphysiol_supplementaryinfo_final_clean_coab021.docx]

Table S1: The lake trout crosses using cold and warm acclimated adults to generate families from parents of similar temperatures (C_♀_xC_♂_, W_♀_xW_♂_) and between temperatures (C_♀_xW_♂_, W_♀_xC_♂_).

|  | Female 1  Cold acclimated | Female 2  Cold acclimated | Female 3  Warm acclimated | Female 4  Warm acclimated |
| --- | --- | --- | --- | --- |
| Male 1  Cold acclimated | Family 1  C_♀_xC_♂_ | Family 5  C_♀_xC_♂_ | Family 9  W_♀_xC_♂_ | Family 13  W_♀_xC_♂_ |
| Male 2  Cold acclimated | Family 2  C_♀_xC_♂_ | Family 6  C_♀_xC_♂_ | Family 10  W_♀_xC_♂_ | Family 14  W_♀_xC_♂_ |
| Male 3  Warm acclimated | Family 3  C_♀_xW_♂_ | Family 7  C_♀_xW_♂_ | Family 11  W_♀_xW_♂_ | Family 15  W_♀_xW_♂_ |
| Male 4  Warm acclimated | Family 4  C_♀_xW_♂_ | Family 8  C_♀_xW_♂_ | Family 12  W_♀_xW_♂_ | Family 16  W_♀_xW_♂_ |

Egg quality measurements

Preliminary analysis was conducted on the lake trout eggs to determine whether egg quality differed between the females of the two different acclimation temperatures. Ten eggs were randomly chosen from each of four cold acclimated females and four warm acclimated females (40 eggs per temperature treatment) and were measured for size and mass on the same day of spawning. Size was measured as diameter (mm) using digital calipers and mass (g) was measured via a microbalance. Water content was determined by measuring the wet and dry mass of an additional 10 eggs from each female. Eggs were pooled per female in aluminum weigh dishes (one dish per female) to measure wet mass, then eggs were placed in an oven at 60℃ for 24 hours to determine dry mass. Water content was expressed as the percentage of egg mass wet weight due to water. To measure energy content, 30 eggs from each female were placed into aluminum weigh dishes, twice rinsed with deionized water to wash away ovarian fluid, then dried in an oven at 60℃ for 24 hours. After drying, eggs were placed into sample tubes and frozen (-20℃) until analysis. Egg energy content was measured in calories per gram of dried eggs via bomb calorimetry with a Parr 6725 semi-micro calorimeter (Parr Instrument Company, Illinois, USA). The means of the mass, size, % water content and energy content between the two temperature treatments were analyzed using student’s t-test in GraphPad Prism 5 (GraphPad Software Inc., La Jolla, California, USA).

Table S2: Measurements of egg quality from cold acclimated (10℃, n = 4) and warm acclimated (17℃, n = 4) lake trout females. Asterix denotes a significant difference between the means (p<0.05).

| **Egg metric** | **Cold acclimated females** | **Warm acclimated females** |
| --- | --- | --- |
| Mass (g) | 0.065 ± 0.001 | 0.059 ± 0.001* |
| Size (mm) | 4.72 ± 0.03 | 4.69 ± 0.03 |
| % Water content | 150.1 ± 2.35 | 150.1 ± 7.88 |
| Energy content (cal/g) | 5703 ± 159 | 5803 ± 58 |

Eggs of warm acclimated female were heavier by 0.006 g, however, no other metrics were significantly different between eggs of cold and warm acclimated females.

DNA extraction and genotyping

Genomic DNA of offspring lake trout was extracted from caudal fin samples, lysing approximately 0.25 cm^2^ of tissue in deep-well 96-well plates by adding approximately 10 mg of tissue to each well along with 250 μL lysis buffer (50 mM Tris pH 8, 1000 mM NaCl, 1 mM EDTA, 1% sodium dodecyl sulphate (SDS) weight per volume, and 1000 μg proteinase K). The plates were incubated for 16 hours at 37°C, after which DNA was precipitated by adding 500 μL of 80% isopropanol per well and centrifuging the plates at 2000 *g* for 45 minutes. Afterwards, the supernatant was removed and the remaining pellets were rinsed with 1 mL of 70% ethanol, followed by re-centrifugation for 45 minutes at 2000 *g*. DNA pellets were air dried in a 70°C incubator for 30 minutes, then dissolved in 150 µl 1x TE (10 mM Tris, 1 mM EDTA). Extraction yields and quality were tested using electrophoresis alongside a mass ladder (Bioshop, Burlington, Ontario) in 1.5% agarose TBE gels stained with Sybr Green (Cedar Lane Laboratories, Burlington, Ontario).

Lake trout DNA samples were amplified at 17 microsatellite loci: MSU01, MSU02, MSU03, MSU05, MSU06, MSU08, MSU09, MSU10, MSU11, MSU13 (Rollins *et al*. 2009), *Ogo*1a (Olsen *et al*. 1998), *Sco*19 (Taylor *et al*. 2001), *Sco*215 (DeHaan *et al*., 2005), *Sfo*1, *Sfo*12 (Angers *et al*. 1995), *Sfo*C88 (King *et al*. 2012), and *Ssa*85 (O'Reilly *et al*. 1996). Multiplex reactions were performed in 10 µl reactions containing the following: 2 µl DNA with approximately 6 ng/µl, 1x PCR buffer containing 1.5 mM MgCl_2_ (Qiagen, Mississauga, Ontario), 2 mM each dNTP (Bioshop, Burlington, Ontario), 0.5 mM MgCl_2_ (Qiagen, Mississauga, Ontario), 0.2 mg/ml BSA (Bioshop, Burlington, Ontario), 0.025 U *Taq* DNA polymerase (Qiagen, Mississauga, Ontario) and ddH_2_O. PCR cycling was carried out on Eppendorf Mastercycler Pro S thermal cyclers. Amplified products for all samples were run on an AB 3730 DNA analysis system with ROX 500 size standard (Applied Biosystems, Foster City, California). Allele sizes were scored using GeneMapper version 3.1 (Applied Biosystems, Foster City, California) and proofread with manual editing.

Sibling relationships were calculated for multilocus genotypes of the offspring using a maximum likelihood relatedness estimator in ML Relate (Kalinowski, Wagner & Taper, 2006). The breeding design (small number of parents, known closed mating history, and equal offspring family sizes) negated the need for more complex analytical approaches, and all offspring were assigned to specific mating crosses with high confidence.

References

Angers, B., Bernatchez, L., Angers, A., L. & Desgroseillers, L. (1995). Specific microsatellite loci for brook charr (*Salvelinus fontinalis*) reveals strong population subdivision on a microgeographic scale. *Journal of Fish Biology,* 47 (Supplement A): 177-185.

Dehaan, P.W., & Ardren, W.R. (2005). Characterization of 20 highly variable tetranucleotide microsatellite loci for bull trout (*Salvelinus confluentus*) and cross‐amplification in other *Salvelinus* species. *Molecular Ecology Notes*, *5*: 582-585.

Kalinowski, S.T, Wagner, A.P., Taper, M.L. (2006). ML-Relate: a computer program for maximum likelihood estimation of relatedness and relationship. *Molecular Ecology Notes* 6: 576-579.

King, T.L., Lubinski, B.A., Burnham-Curtis, M.K., Stott, W., & Morgan, R.P. (2012). Tools for the management and conservation of genetic diversity in brook trout (*Salvelinus fontinalis*): tri-and tetranucleotide microsatellite markers for the assessment of genetic diversity, phylogeography, and historical demographics. *Conservation Genetics Resources*, *4*: 539-543.

Olsen, J. B., Bentzen, P., & Seeb, J. E. (1998). Characterization of seven microsatellite loci derived from pink salmon. *Molecular Ecology*, 7: 1087-1090.

O'Reilly, P.T., Hamilton, L.C., McConnell, S.K., & Wright, J.W. (1996). Rapid analysis of genetic variation in Atlantic salmon (*Salmo salar*) by PCR multiplexing of dinucleotide and tetranucleotide microsatellites. *Canadian Journal of Fisheries and Aquatic Sciences,* 53: 2292-2298.

Rollins, M.F., Vu, N.V., Spies, I.B., & Kalinowski, S.T. (2009). Twelve microsatellite loci for lake trout (*Salvelinus namaycush*). *Molecular Ecology Resources,* 9: 871-873.

Taylor, E.B., Redenbach, Z.A., Costello, A.B., Pollard, S.J., & Pacas, C.J. (2001). Nested analysis of genetic diversity in northwestern North American char, Dolly Varden (*Salvelinus malma*) and bull trout (*Salvelinus confluentus*). *Canadian Journal of Fisheries and Aquatic Sciences,* 58: 406-420.
